# Incorporation of Poly(Itaconic Acid) with Quaternized Thiazole Groups on Gelatin-Based Films for Antimicrobial-Active Food Packaging

**DOI:** 10.3390/polym13020200

**Published:** 2021-01-08

**Authors:** Celeste Cottet, Andrés G. Salvay, Mercedes A. Peltzer, Marta Fernández-García

**Affiliations:** 1 Laboratory of Obtention, Modification, Characterization and Evaluation of Materials (LOMCEM), Department of Science and Technology, University of Quilmes, Roque Sáenz Peña 352, Bernal B1876BXD, Buenos Aires, Argentina; ccottet@uvq.edu.ar (C.C.); asalvay@unq.edu.ar (A.G.S.); 2 Scientific Research Commission (CIC), 526 st, La Plata B1900, Buenos Aires, Argentina; 3 National Scientific and Technical Research Council (CONICET), Godoy Cruz 2290, (C1425FQB) Ciudad Autónoma de Buenos Aires, Argentina; 4 Macromolecular Engineering Group, Institute of Polymer Science and Technology, (ICTP-CSIC), Juan de la Cierva 3, 28006 Madrid, Spain; 5 Interdisciplinary Platform for Sustainable Plastics towards a Circular Economy, SUSPLAST, CSIC, 28006 Madrid, Spain

**Keywords:** poly(itaconic acid), quaternized polymers, biodegradable polymers, antimicrobial activity, antioxidant activity, active packaging

## Abstract

Poly(itaconic acid) (PIA) was synthesized via conventional radical polymerization. Then, functionalization of PIA was carried out by an esterification reaction with the heterocyclic groups of 1,3-thiazole and posterior quaternization by N-alkylation reaction with iodomethane. The modifications were confirmed by Fourier transform infrared (FTIR) and proton nuclear magnetic resonance (^1^H-NMR), as well as ζ-potential measurements. Their antimicrobial activity was tested against different Gram-negative and Gram-positive bacteria. After characterization, the resulting polymers were incorporated into gelatin with oxidized starch and glycerol as film adjuvants, and dopamine as crosslinking agent, to develop antimicrobial-active films. The addition of quaternized polymers not only improved the mechanical properties of gelatin formulations, but also decreased the solution absorption capacity during the swelling process. However, the incorporation of synthesized polymers increased the deformation at break values and the water vapor permeability of films. The antioxidant capacity of films was confirmed by radical scavenging ability and, additionally, those films exhibited antimicrobial activity. Therefore, these films can be considered as good candidates for active packaging, ensuring a constant concentration of the active compound on the surface of the food, increasing products’ shelf-life and reducing the environmental impact generated by plastics of petrochemical origin.

## 1. Introduction

Nowadays, consumers’ demands are focused on the innovations in food packaging technologies [1]. The challenge is to produce materials and packaging that increase food shelf-life, keeping food safer and healthier, under the current legislation. Active films are those that, in addition to protecting food passively against mechanical stress or preventing dehydration or oxidation thanks to their mere presence, contain compounds that may be released to the food, extending its useful life while maintaining its quality attributes [2,3,4]. Polymeric antimicrobial agents in active systems provide many advantages regarding food preservation, as the addition of additives in their formulation is avoided and a constant concentration of active compound is ensured on the surface of the food, where contamination mainly takes place [5]. These systems inhibit or reduce bacterial growth (antimicrobial), and/or eliminate or hinder bacterial adhesion (antifouling) [6]. Commonly used antimicrobial agents are chemically produced or extracted from biomass of animals, plants, and microorganisms, including chitosan, enzymes, essential oils, and natural extracts from different plant sources [7]. The manufacture of new polymeric materials with antimicrobial and/or antifouling capacity has been postulated as a very attractive alternative [8,9,10,11,12,13]. Because of their high molecular weight and low diffusion coefficient, antimicrobial polymers present limited leaching from surfaces where they are incorporated [13,14]. Recently, research related to this topic has been conducted on a wide variety of antimicrobial polymeric systems including quaternary ammonium compounds, polymeric quaternary phosphonium salts, guanidine-containing polymers, and halogen polymers (i.e., N-halamines), among others [9,15]. Most of the systems studied are polycations, in particular, they present quaternary nitrogen atoms [11,16]. Their mechanism of action is not very well understood, but polycationic structures interact electrostatically with negatively charged bacterial membrane, breaking the rupture of cell wall and subsequent death of the microorganism.

Because of the increasing interest in sustainable development, organic acids like itaconic acid (IA), which is an unsaturated dicarbonic organic acid, have gained considerable attention [17,18]. IA is a natural acid produced industrially by biochemical processes through the fermentation of sugars. Thanks to biotechnological advances in the efficiency of its production, the price of IA has decreased and, therefore, interest in its use in the biobased plastics industry has grown considerably [19,20,21]. It is considered as one of the twelve most important biomass-derived compounds that can be transformed into a wide range of valuable chemicals or materials. Studies have shown that persulphate-initiated polymerization of the acid in aqueous media makes it possible to prepare itaconic polymers via initiator-free radical polymerization [22].

The novelty of the present work was to use a biobased system to obtain a cationic polymer. It is known that quaternary ammonium bactericides (QAS) are systems that migrate easily, which is not ideal for food packaging [23]. On the contrary, polymeric-cationic systems of high molecular weight do not present the problem of migration to the surrounding media. The use of thiazole groups as antimicrobial was previously used with excellent results [24,25,26], and the interest of using it in biobased monomers such as itaconic acid gives an added value to this work.

In addition, biopolymers are well-known renewable source-based materials, which are environmentally benign, with increasing importance for different applications [10,27,28,29,30]. Among the natural polymers available to form films, starch and gelatin appear as potential sources to replace widely used polymers of petrochemical origin [31,32,33,34].

Starch is a polysaccharide and one of the most promising biopolymers because it is easily available, biodegradable, and has a low cost [29]. However, starch presents weaknesses including poor solubility, low mechanical properties, and instability at high temperature and pH during processing. To overcome these limitations, various chemical modifications of the hydroxyl groups in the anhydroglucose units have been proposed [35].

Likewise, gelatin is a high molecular weight polypeptide and an important hydrocolloid in the food industry owing to its inherent characteristics. It offers a wide range of further and unique industrial applications and is very valuable as a biopolymer for material developments [32,36]. However, gelatin films presented poor mechanical properties, which limit its application as a packaging material; thus, structural modifications are required to enhance its mechanical strength. Several studies have demonstrated that gelatin can be used together with starch as reinforcement to the polymeric matrix [37]. In this sense, different authors have investigated gelatin and starch mixtures in different proportions to develop edible films with improved properties [38]. Moreover, natural phenolic compounds as crosslinkers have shown great efficiency in protein modifications [10,39]. Many phenolic compounds have been reported to be interactive with proteins and resulted in improved film properties for gelatin-based materials [33]. Among them, dopamine is a well-known cross-linker for gelatin-based materials [40,41]. Previous studies also demonstrated that dopamine has antioxidant activity, which is stronger than other related compounds, correlated to the number of hydroxyl groups on the phenolic ring [42]. The incorporation of these agents into polymeric materials will develop antioxidant systems, expanding the functionality of the packaging by adding an extra function.

The present work has as a main objective the development of antimicrobial polymers obtained by chemical modification of poly(itaconic acid) incorporating thiazole groups and posterior quaternization. In addition, the incorporation on gelatin-based films for antimicrobial-active food packaging was studied and characterized.

## 2. Materials and Methods

### 2.1. Materials

Itaconic acid (IA, Sigma-Aldrich^®^, Darmstadt, Germany), ammonium persulfate (Sigma-Aldrich^®^), acetone (≥99.5, Sigma-Aldrich^®^), 4-methyl-5-thiazoleethanol (Sigma-Aldrich^®^), sulfuric acid (99%, Sigma-Aldrich^®^), iodomethane (99%, Sigma-Aldrich^®^), anhydrous N,N-dimethylformamide (DMF, 99.8%, Sigma-Aldrich^®^), n-hexane (99%, Sigma-Aldrich^®^), dimethyl sulfoxide (DMSO, >99%, Alfa Aesar^®^, Kandel, Germany), cellulose membrane D80 (Orange Scientific^®^, Braine-l’Alleud, Belgium), deuterated DMSO solvent (DMSO-d6, Sigma-Aldrich^®^), gelatin (Scharlau^®^), starch (Nestlé Health Science^®^, Barcelona, Spain), glycerol (≥99%, Sigma-Aldrich^®^), dopamine hydrochloride (99%, Alfa Aesar™), sodium hydroxide (NaOH, ≥98%, Sigma-Aldrich^®^), potassium carbonate (K_2_CO_3_, Sigma-Aldrich^®^), magnesium nitrate (Mg(NO_3_)_2_, 98%, Sigma-Aldrich^®^), phosphate buffer solution (PBS, pH 7.4, Sigma-Aldrich^®^), 2,2′-azino-bis(3-ethylbenzothiazoline-6-sulfonic acid) (ABTS, Sigma^®^ A1888), potassium persulfate (K_2_S_2_O_8_, Sigma^®^ 216224, Saint Louis, MO, USA), ethanol 96% vol. (Porta^®^), peptone (Britania^®^, CABA, Argentina), bacteriological agar (Britania^®^), Mueller–Hinton broth (Britania^®^), nutritive broth (Britania^®^), and sterile 96-well microplate (AP-Biotech^®^, Buenos Aires, Argentina) were used as received.

### 2.2. Synthesis of Quaternized Polymers, MeFPIAx

**Scheme 1:**
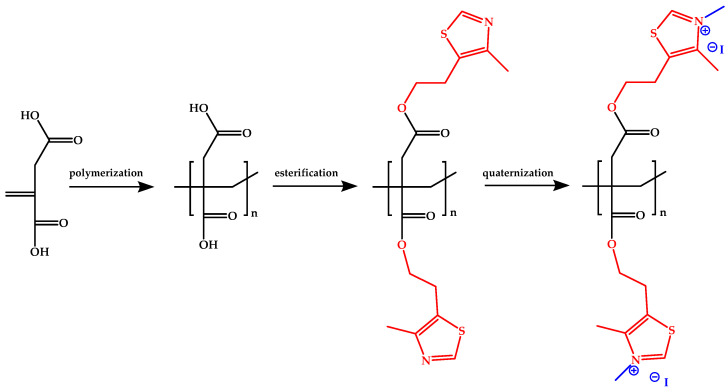
Different steps of the quaternized polymers’ synthesis.

Scheme 1 represents the different steps of the quaternized polymers’ synthesis.

#### 2.2.1. Synthesis of Poly(Itaconic Acid) (PIA)

Firstly, PIA homopolymer was synthesized via conventional radical polymerization of itaconic acid [43]. IA (0.023 mol) and the initiator ammonium persulfate (2.22 × 10^−4^ mol) were dissolved in distilled water (10 mL) in a glass tube. The mixture was continuously stirred under an argon bubbling atmosphere (only the first hour) in a thermostatic bath at 60 °C for 48 h. Initially, the reaction mixture obtained was heterogeneous bevause of an incomplete dissolution of IA, but after 5 min, it turned transparent. After the reaction time was completed, the tube was removed and cooled down at room temperature, also under continuous stirring. The obtained polymer, PIA, was separated from the reaction mixture by precipitation in cool acetone under vigorous stirring. The precipitate formed was separated by filtration and dried under vacuum at room temperature until constant weight.

#### 2.2.2. Functionalization of PIA with 1,3-Thiazole Side-Chain Groups (FPIAx)

The functionalization of PIA was carried out by an esterification reaction with the heterocyclic groups of 1,3-thiazole. PIA (0.015 mol) with an excessive amount of 4-methyl-5-thiazoleethanol (8 mL) (molar relationship 1:4) were added into a sealed tube and placed in a thermostatic bath at 90 °C and stirred until complete dissolution. Then, 0.012 mL of sulfuric acid was added as a catalyst and the reaction was continued for 24 h at 90 °C. After this period, the system was cooled down at room temperature. Then, the functionalized polymer (FPIA1) was removed from the reaction mixture by precipitation in cool water under vigorous stirring. The precipitate formed was separated by filtration and dried under vacuum at room temperature until constant weight. On the other hand, another PIA functionalization was performed, but instead of 90 °C, the reaction was carried out at 120 °C (FPIA2).

#### 2.2.3. Quaternization of FPIAx with Iodomethane (MeFPIAx)

FPIA1 and FPIA2 polymers were modified by *N*-alkylation reaction with iodomethane. FPIAx with an excess of iodomethane (molar relationship 1:10) were dissolved in the minimum necessary amount of anhydrous *N*,*N*-dimethylformamide in a sealed tube. The system was placed in a thermostatic bath at 70 °C and stirred for 7 days. After the reaction was completed and cooled down, the polymers were separated by precipitation in a solution of n-hexane under vigorous stirring, until a phase separation was produced. The polar phase was diluted with DMSO/H_2_O 2/1 and purified by dialysis against distilled water for 7 days (cellulose membrane with a molecular weight cut off 6000–8000). Finally, quaternized polymers (MeFPIA1 and MeFPIA2) were obtained after freeze-drying under vacuum.

### 2.3. Physicochemical Characterization of Synthesized Polymers

Proton nuclear magnetic resonance (^1^H-NMR) spectra were made on a Bruker 400 MHz NMR spectrometer (Bruker, Madrid, Spain) in DMSO-d6 at room temperature. Attenuated total reflectance Fourier transformed infrared (ATR-FTIR) spectra were performed on a Perkin Elmer RX-1 instrument (Boston, MA, USA). Gel permeation chromatography (GPC) was performed to determine the number average molecular weight and polydispersity index (M_n_, and PI) of the polymer in water with NaNO_2_ as eluent at 1 mL/min flow and 30 °C in a chromatographic system with a Perkin Elmer Isocratic LC pump 250 and a refraction index detector (Series 200, BostonMA, USA). Ultrahydrogel^TM^ HR column (Waters Division Millipore, Milford, MA, USA) was used. Pullulan standards (Sigma Aldrich) were used to calibrate the columns. The ζ potential of cationic polymers was obtained with a Zetasizer Nano series ZS (Malvern Instrument Ltd., Malvern, UK), equipped with an He-Ne laser beam at 658 nm. The data were averaged over at least five runs.

### 2.4. Preparation of Active Films Based on Gelatin and Starch

Aqueous dispersions at 30% *w*/*w* (referring to the total of components) based on gelatin and starch (4:1 mass ratio) were prepared in distilled water. Then, 10% *w*/*w* of the synthesized polymers (MeFPIA1 and MeFPIA2) regarding weight of gelatin and starch was added. Glycerol 50% *w*/*w* was added based on the dry weight of the total of polymers. A control sample without the addition of the synthesized polymers was also prepared. Dopamine hydrochloride at 2% *w*/*w* regarding weight of the total of polymers was incorporated into half of the formulations. Dopamine was incorporated to interact with the polymers and provide more stability to the films and antioxidant activity [42]. Dispersions composed of gelatin, starch, glycerol, and the synthesized polymers were stirred in a thermostatic bath at 70 °C for 20 min, and the pH of the solutions was adjusted to a target value (8.5–9) by the addition of NaOH 0.1 M. After adjusting the pH, dopamine was added and left stirring in the thermostatic bath (70 °C) for another 20 min. Finally, films were casted in 90 mm diameter Petri dishes and dried for one week at room temperature. The dried films were peeled from the plates and conditioned for one week at 43% relative humidity (r.h.) provided by a saturated solution of K_2_CO_3_ and 25 °C. The thickness of the films obtained was 0.18 ± 0.02 mm.

### 2.5. Mechanical Properties

Mechanical properties were tested at room temperature using a Universal Test Instrument Megatest TC-500 series II (Megatest, Argentina) equipped with a 30 kgf cell load, and experiments were performed at 10 mm/min. Before testing, rectangular samples (dimensions 50 mm × 10 mm) were cut for each formulation, and conditioned at 53% r.h. provided by a saturated solution of Mg(NO_3_)_2_. The thickness of each rectangle was determined at five points using a digital micrometer (INSIZE CO., LTD, Suzhou New District, China, ±0.001 mm). The resulting stress–strain curves allowed calculating the mechanical parameters: Young’s modulus (YM, MPa), tensile strength (TS, MPa), and elongation at break (EB, %).

### 2.6. Color Properties

Cielab coordinates were recorded using a Konica Minolta CR400 spectrophotometer (Tuscaloosa, NJ, USA) to evaluate the appearance of the films. L* corresponds to the luminosity or darkness of a color, a* value corresponds to the green to red color (−a = greener, +a = redder), and b* to the blue to yellow (−b = bluer, +b = yellower). The color change ΔE was also calculated through Equation (1).

(1) $$\Delta E=\sqrt{{\left(a^{*}-a_{0}^{*}\right)}^{2}+{\left(b^{*}-b_{0}^{*}\right)}^{2}+{\left(L^{*}-L_{0}^{*}\right)}^{2}}$$

where *a*_0_*, *b*_0_*, and *L*_0_* are the coordinates corresponding to the control film, against which the rest of the films were compared in order to determine the color change owing to the synthesized polymers and/or dopamine.

### 2.7. Experimental Water Vapor Permeability (P_w_^exp^)

*P_w_^exp^* of the films was measured using the gravimetric technique of ASTM E-96 [44] with some modifications [45]. The permeability was obtained by quantifying the flow of water vapor through the film by changes in weight of the system, owing to moisture transfer. Firstly, films were sealed on acrylic cups with a 53 mm diameter aperture, containing a saturated solution of BaCl_2_, which generates a relative humidity of 90%. Test cups were placed inside a desiccator containing a saturated solution of NaOH to provide a relative humidity of 10%, at a constant temperature of 22 °C. A fan was placed over the films to maintain uniform conditions inside the desiccator. Cup weights were recorded at the beginning of the experiment using an analytical precision balance (Precisa 125 A SCS, ±10^−4^ g), and then weight loss was measured over time. Weight loss *m* versus time *t* was plotted and, when the steady state was reached (straight line), another 48 h were registered. *P_w_^exp^* (g·s^−1^·m^−1^·Pa^−1^) was calculated according to Equation (2).

(2) $$P_{w}^{exp}=\frac{1}{A}\left(\frac{\Delta m}{\Delta t}\right)\frac{L}{\Delta p_{w}}$$

where *A* is the effective area of the exposed film and ∆m/∆t is the slope of a linear regression of weight loss versus time. L is the film thickness and Δ*p_w_* = (*p_w_*_2_ − *p_w_*_1_) (in Pa units) is the differential water vapor partial pressure across the film; *p_w_*_1_ is the partial pressure of water vapor at the film surface outside the cup (263.9 Pa); and *p_w_*_2_ is the partial pressure of water vapor at the film surface inside the cup (2375.4 Pa). Experiments were performed in triplicate.

### 2.8. Swelling Properties

#### Evaluation of Swelling Capacity of Films and Their Kinetics

Swelling of polymeric films was evaluated in phosphate buffer solution (PBS, pH 7.4) at 25 and 37 °C. Film discs were weighed (about 40 mg) and immersed in containers with 20 mL of the buffer. Swelling was followed gravimetrically, measuring the solution gain over the immersion time. At a certain time, the discs were removed and the excess of solution was carefully eliminated with a filter paper, weighed, and returned again to the containers. This procedure was performed until there was no more solution gaining from the film (constant weight) or loss of integrity. Three replicates of each film were tested. The swelling process was expressed as solution uptake, *h*(*t*), at time t in units of g of solution per 100 g dried film (d.f.), and was calculated by Equation (3).

(3) $$h\left(t\right)=\;\frac{W_{t}-W_{0}}{W_{0}}\times 100$$

where *W_t_* is the weight of the swollen disc at time *t* and *W*_0_ is the weight of the conditioned disc at the beginning of the experiment.

The solution uptake h as function of time *t* was fitted with a first-order kinetics model [46], using a biexponential function that takes into account two rates for solution uptake [47], as displayed in Equation (4).

(4) $$h\left(t\right)=h_{0}+\Delta h_{1}(1-\left(1-exp\left(-t/\tau_{1}\right)\right)+\Delta h_{2}\left(1-exp\left(-t/\tau_{2}\right)\right)$$

where *h*(*t*) is the solution uptake; *h_0_* is the initial solution content (*h*_0_ = 0 in the experimental conditions); Δ*h*_1_ and Δ*h*_2_ are the solution uptake related to sorption process 1 and 2, respectively; and *τ*_1_ and *τ*_2_ are the time constants for process 1 and 2, respectively. The solution uptake at equilibrium *h*_∞_ was calculated through Equation (5).

(5) $$h_{\infty }=\Delta h_{1}+\Delta h_{2}$$

### 2.9. Evaluation of the Antioxidant Properties by the ABST^•+^ Assay

#### 2.9.1. Preparation of Working Solutions 

The antioxidant activity of the films was evaluated applying the ABTS method. For this, ABTS was prepared in aqueous solution in a concentration of 7 mM and mixed with K_2_S_2_O_8_ to reach 2.45 mM concentration in order to form the radical ABTS^•+^ (solution A). This mixture was left for 16 h in the dark, and after this period of time, solution A was diluted in different solvents: in milliQ water (solution B), ethanol/water 50/50 (solution B_1_) and ethanol/water 95/5 (solution B_2_), until the absorbance of the solutions was adjusted to 0.70 ± 0.02 at a wavelength of 734 nm.

#### 2.9.2. Estimation of Film Antioxidant Capacity by Direct Contact with ABTS^•+^

Discs of about 10 mg of each sample were cut and placed in eppendorf tubes with 1 mL of the corresponding B_n_ solution. After 30 min of reaction in the dark with slight agitation, the absorbance of the solution was measured at a wavelength of 734 nm. Each condition was observed at least in duplicate. The antioxidant activity of the different films was calculated by the radical scavenging activity percentage (*RSA*(%)), as described in Equation (6).

(6) $$RSA\left(\%\right)=\frac{blank\;absorbance-sample\;absorbance}{blank\;absorbance}\times 100$$

where blank corresponds to ABTS solution (B_n_) absorbance without film.

### 2.10. Microbiological Assays

#### 2.10.1. Minimal Inhibition Concentration (MIC) of MeFPIAx Quaternized Polymers

MICs were tested by broth microdilution methods against Gram-positive bacteria *Staphylococcus aureus* (ATCC 25923) and Gram-negative *Escherichia coli* (ATCC 25922) and *Pseudomonas aeruginosa* (ATCC 9027) bacteria strains obtained by Thermo Scientific™, according to the guidelines of the Clinical Laboratory Standards Institute [48].

A microorganism suspension of ~10^8^ colony forming units (CFU)/mL in sterile peptone water 0.1% was prepared to obtain a turbidity equivalent of 0.5–1.0 McFarland opacity standard. Previously, bacterial isolates were cultured on nutrient agar for 24 h at 37 °C. This suspension was diluted 1:100 in Mueller–Hinton broth to achieve an inoculum suspension of ~10^6^ CFU/mL. Moreover, each polymer was dissolved in sterile distilled water to yield a stock solution of 32 mg/mL. Then, 200 μL of polymer stock solution was pipetted into the first column of a sterile 96-well microplate and 100 μL of Mueller–Hinton broth was added into the rest of the wells, except in the first column (already filled with the stock solution). Then, 100 μL of polymer stock solution was diluted by twofold serial dilutions in the rest of the wells, except in the last column. Finally, 100 μL of each test microorganism suspension was inoculated into all the wells, affording 200 μL of final polymer concentrations of 16, 8, 4, 2, 1, 0.5, 0.25, 0.125, 0.063, 0.031, and 0.016 mg/mL. The last column of wells, which did not contain polymer, was used as positive growth control of microorganisms. Microplates were incubated at 37 °C for 24 h; after this period of time, MIC was defined visually as the lowest polymer concentration at which growth of the microorganism was inhibited.

#### 2.10.2. Antimicrobial Activity of the Films

The methodology developed was based on the ASTM E2149 [49]. The antimicrobial activity of the films was evaluated against Gram-positive *S. aureus* bacteria strains (ATCC 25923). The microorganism isolate was incubated in sterile nutrient agar plates for 24 h at 37 °C. Then, a microorganism suspension was prepared in tubes with 0.1% sterile peptone water until a turbidity equivalent of 0.5 McFarland (10^8^ CFU/mL). This inoculum suspension was diluted into sterile PBS (pH 7.4) to obtain the inoculum final concentration of 10^5^ CFU/mL. Before testing, the films were conditioned at 53% r.h. Then, samples were cut into squares of 1 cm^2^ and placed in eppendorf tubes with 750 µL of the inoculum solution and 750 µL of PBS. They were incubated at 37 °C while shaking at 100 rpm. Aliquots were taken and total cell counts were made at 0 and 24 h of contact time. A control of the inoculum solution was carried out (without film) and those that did not contain MeFPIAx were also considered as control films. The percentage of bacterial reduction (%) was calculated using Equation (7).

(7) $$Bacterial\;reduction\;\left(\%\right)\;=\frac{B-A}{B}\times 100$$

where *A* is CFU/mL for the film after 24 h contact time, and *B* is CFU/mL at the initial time.

### 2.11. Statistical Analysis

Data were expressed as means ± S.D. and were analyzed by analysis of variance (ANOVA) tests using PSPP Software. Tukey’s honest significance test was applied to find means that were significantly different from each other, with a significance level of *p* < 0.05.

## 3. Results and Discussion

### 3.1. Homopolymer Synthesis and Modification

**Figure 1:**
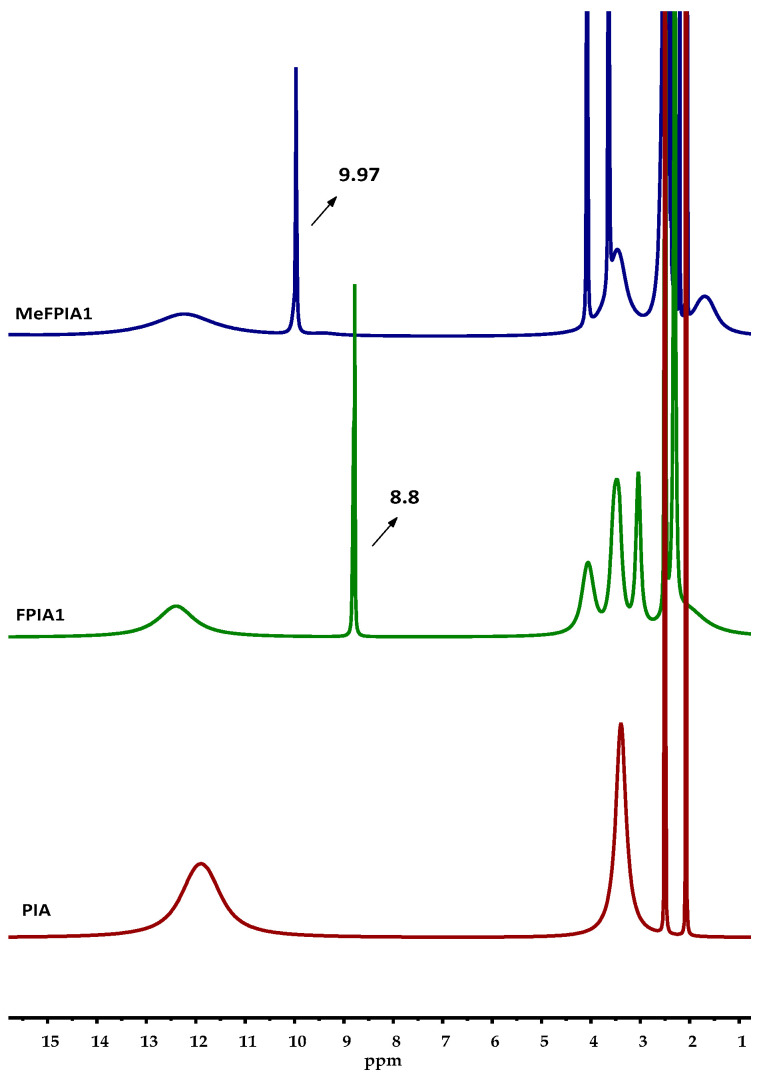
Proton nuclear magnetic resonance (^1^H-NMR) spectra of poly(itaconic acid) (PIA), functionalized polymer (FPIA1), and quaternized polymer (MeFPIA1).

PIA was synthesized in water at 60 °C, as described previously. The yield was 60% and the number average molecular weight (MW) and its polydispersity index were 59700 Da and 1.9, respectively. It is important to mention that the relationship between hydrodynamic volume and molecular weight in the polymer is different from the standards. Therefore, these data should be taken as an estimation of MW. Figure 1 shows the proton spectrum of PIA, where methylene protons at 2.0 ppm corresponding to the main chain and methylene protons at 3.5 ppm assigned to –CH_2_–COOH are easily identified.

Modified polymers MeFPIAx were prepared at two different temperatures (90 and 120 °C). The characteristic peaks are identified in the figure; the CH_2_ of the main chain at 2.3 ppm and the methylene protons of –CH_2_–COO– at 3.5 ppm are slightly shifted to higher displacement as a result of the higher rigidity. The methylene protons near to ester (O–CH_2_–) appear at 4.0 ppm, while the CH_2_ protons bonded to the thiazole group appear at 3.0 ppm. The CH_3_ protons of thiazole group emerge at 2.3 ppm along with the methylene protons of the main chain. The degree of modification was determined by ^1^H-NMR spectroscopy by comparison of triazole protons at 8.8 ppm and the methylene protons of –CH_2_–COO–. The results were 28% and 60% for FPIA1 and FPIA2, respectively. The increment in the temperature produces a higher degree of modification. After that, the total quaternization of the pendant heterocycles groups was achieved in both cases. There are no signals at ca. 8.8 ppm corresponding to thiazol protons and new peaks appear at ca. 10 ppm from the protons of both thiazolium groups.

**Figure 2:**
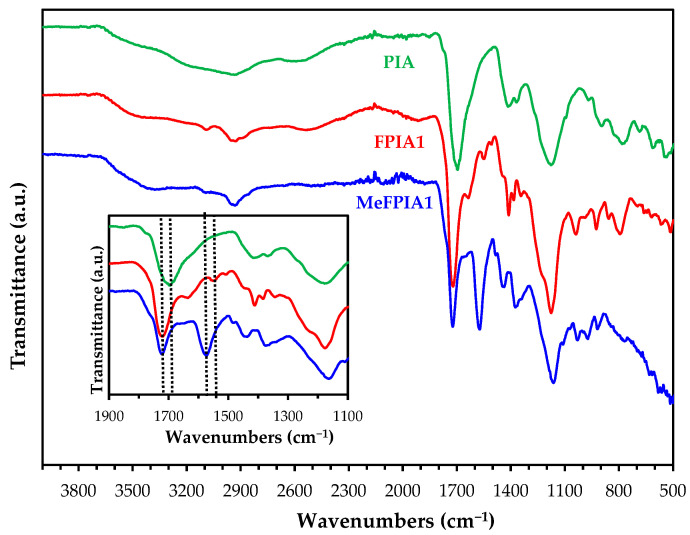
Fourier transform infrared (FTIR) spectra of PIA, modified FPIA1, and quaternized MeFPIA1 polymer.

Figure 2 displays, as an example, the FTIR spectra of PIA, FPIA1, and MeFPIA1. PIA homopolymer spectrum shows one characteristic band at 1695 cm^−1^ (C=O), which should be assigned to the carboxyl group. The spectrum of FPIA1, polymer with lower degree of modification, shows other characteristics bands, such as the following: 3082 cm^−1^ (=C–H thiazole), 2928 cm^−1^ (C–H), 1720 cm^−1^ (C=O), 1547 cm^−1^ (C=N thiazole), and 1117 cm^−1^ (C–O) [24]. Considering these signals, it could be concluded that the functionalization of PIA was achieved. Additionally, it could be seen in the MeFPIA1 spectrum that the band corresponding to the C=N bond was displaced at 1572 cm^−1^ (C=N^+^ thiazolium) owing to the quaternization.

Additionally, ζ-potential values for charged polymers were determined. Measurements were made from solutions of 1 mg/mL of polymer in MilliQ water (18 mΩ). The values were −60 mV for PIA, −52 mV for MeFPIA1, and −9 mV for MeFPIA2. The negative ζ-potential observed in PIA due to the presence of carboxylate groups was shifted to less negative as the degree of modification increases. Therefore, the results confirm that a greater degree of modification was obtained with the increasing reaction temperature, as MeFPIA2 presented higher ζ-potential than MeFPIA1. These negative values were changed with the addition of a few drops of hydrochloric acid solution 0.1 M, where the ζ-potential of the polymers presented less negative values, as the free carboxylic groups were protonated. Therefore, both MeFPIA homopolymers have a dual-nature, and the presence of positive and negative charges along the side chain can produce some variations in conformation, giving the possibility to be considered as zwitterionic polymers [50]. These zwitterions, including polyampholytes and polybetaines, are macromolecules with oppositely charged groups (cationic and anionic) along the chain or side chain that show different behaviors depending on their environment. Considering this definition, the charges could be distributed throughout the chain in different ways [51] and, in this case, is a random distribution. Typically, cation groups are quaternized ammonium, and anionic groups are sulfonates, carboxylates, and phosphonates [52]. The chain’s ionization depends on the pH of the environment, which has a marked effect on the polymer’s conformational behavior. The use of a carboxylate as the anion at acidic environments resulted in the protonation and neutralization of the monomer units [51]. Zwitterionic polymers have great potential as anti-fouling coating, between other functionalities, because they can resist nonspecific protein adsorption, bacterial adhesion, and biofilm formation [53].

After the polymers were characterized, they were incorporated into gelatin- and starch-based films with glycerol as plasticizer. Half of the formulations made incorporated dopamine as a crosslinking agent to improve their properties. Table 1 shows the names and the description of each film formulation; those without polymer and dopamine were considered as control films.

### 3.2. Mechanical Properties

The tensile test results are presented in Table 2, and the increase in TS and EB was observed with the addition of MeFPIAx (GS-MeFPIA1 and GS-MeFPIA2), showing a statistically significant variation (*p* < 0.05). Gelatin is a very valuable biopolymer; however, its poor mechanical properties limit its application as a packaging material [54]. The considerable improvement in TS and EB may be due to the presence of increasing electrostatic forces and hydrogen bonding interactions between the gelatin macromolecules and carboxyl groups of MeFPIAx, constituting a stable network with enhanced mechanical properties [55].

The difference between the films with both functionalized polymers is that MeFPIA1 had a greater number of free carboxyl groups capable of interacting with the protein chains. Gelatin has an amphoteric character because of the functional groups of the amino acids and the terminal amino and carboxyl groups created during collagen hydrolysis [56], so they are able to form links with polymer chains. No differences were observed on YM values.

Similar results were obtained with the incorporation of dopamine into the mixture. Significant differences (*p* < 0.05) were found in films GS-MeFPIA1D and GS-MeFPIA2D compared with the control sample with only dopamine without modified polymers (Gel-D). GS-MeFPIA1D and GS-MeFPIA2D films showed higher values of EB, probably owing to the increase of interactions generated by both polymers (as mentioned above) and dopamine added to form network points [57]. Dopamine belongs to the catechol family and its incorporation in almost any polymer matrix induces the reaction with the functional groups on the polymer backbone in a weak alkaline environment [55]. Although there was a slight increase in TS, as expected, this difference was not significant. Moreover, a decrease in YM was observed. This result could be explained as dopamine may interfere between strong interactions between gelatin and polymer chains.

### 3.3. Color Properties

**Figure 3:**
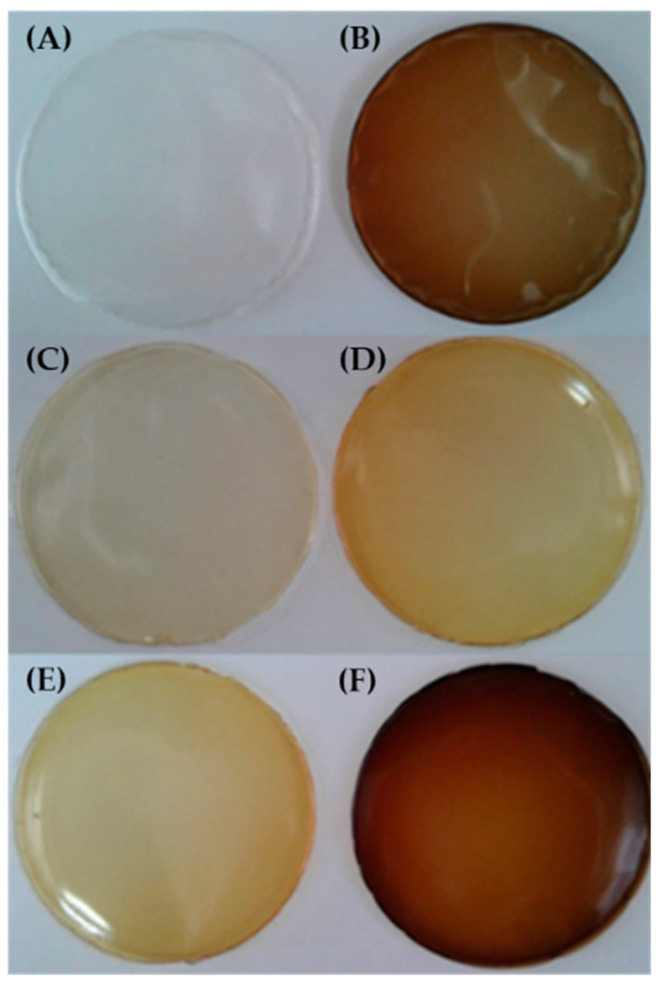
Photographs of the different films: (**A**) GS, (**B**) GS-D, (**C**) GS-MeFPIA1, (**D**) GS-MeFPIA1D, (**E**) GS-MeFPIA2, and (**F**) GS-MeFPIA2D.

Color properties are shown in Table 3 and a clear change in color values was observed with the addition of MeFPIAx. The presence of MeFPIA2 in the formulation caused a decrease in the luminosity (decrease in L* values) of the samples. Moreover, films with MeFPIA1 and MeFPIA2 turned greenish (decrease in a* values) and yellowish (increase in b* values), the most pronounced increase in the yellow color of the films was observed with the addition of MeFPIA2. Therefore, the color change ΔE* is significant for both polymers and perceptible to the human eye, as can be seen in Figure 3; this change was more abrupt for the polymer with less negative potential.

Moreover, the addition of dopamine produced a change even greater in all the parameters studied. The L* value of samples was significantly reduced. Samples with dopamine tend to be reddish (increase in a* values), even more in the sample with MeFPIA2. Moreover, an increase in b* values towards yellow was observed in all the samples.

Generally, colorless films are desirable for food packaging, but sometimes, transparency could be a drawback owing to the light exposure, causing food deterioration. Therefore, films with such a color could protect food from light degradation [58]. In this way, the incorporation of MeFPIAx could be advantageous.

### 3.4. Experimental Water Vapor Permeability (P_w_^exp^)

The quality of most food products deteriorates via moisture absorption, and this can occur between food and the atmospheric environment, so it is important to characterize the barrier properties of films against water vapor [59]. The flow of water through polymeric films does not occur through pores, but can be understood considering that the process occurs in three stages: (i) sorption of water vapor at the surface layer of the film in contact with the highest internal r.h.; (ii) diffusion of the permeant molecules through the film; and (ii) desorption of water vapor from the other surface of the film, where the lowest external r.h. occurs [60]. Hence, the chemical structure, polarity, degree of crystallinity, density, crosslinking degree, molecular weight, and polymerization, as well as the presence of plasticizers, are factors to take into account because they will affect water vapor permeability [45].

As can be seen in Table 4, the incorporation of MeFPIAx caused an increase in *P_w_^exp^*. Considering the mechanical properties, it was observed that the incorporation of polymer increased the elongation of the materials; therefore, as the polymeric chains have more mobility, the passage of water vapor through the films is facilitated [61]. Moreover, when samples with MeFPIAx are compared to films with MeFPIAx and dopamine, it could be seen that dopamine incorporation slightly reduced the *P_w_^exp^* values. This could be explained with the fact that dopamine might increase polymer chains’ interactions, making the passage of water vapor more difficult. This effect was not so significant in those films that do not contain MeFPIAx, as the number of interactions was lower.

### 3.5. Swelling Properties

**Figure 4:**
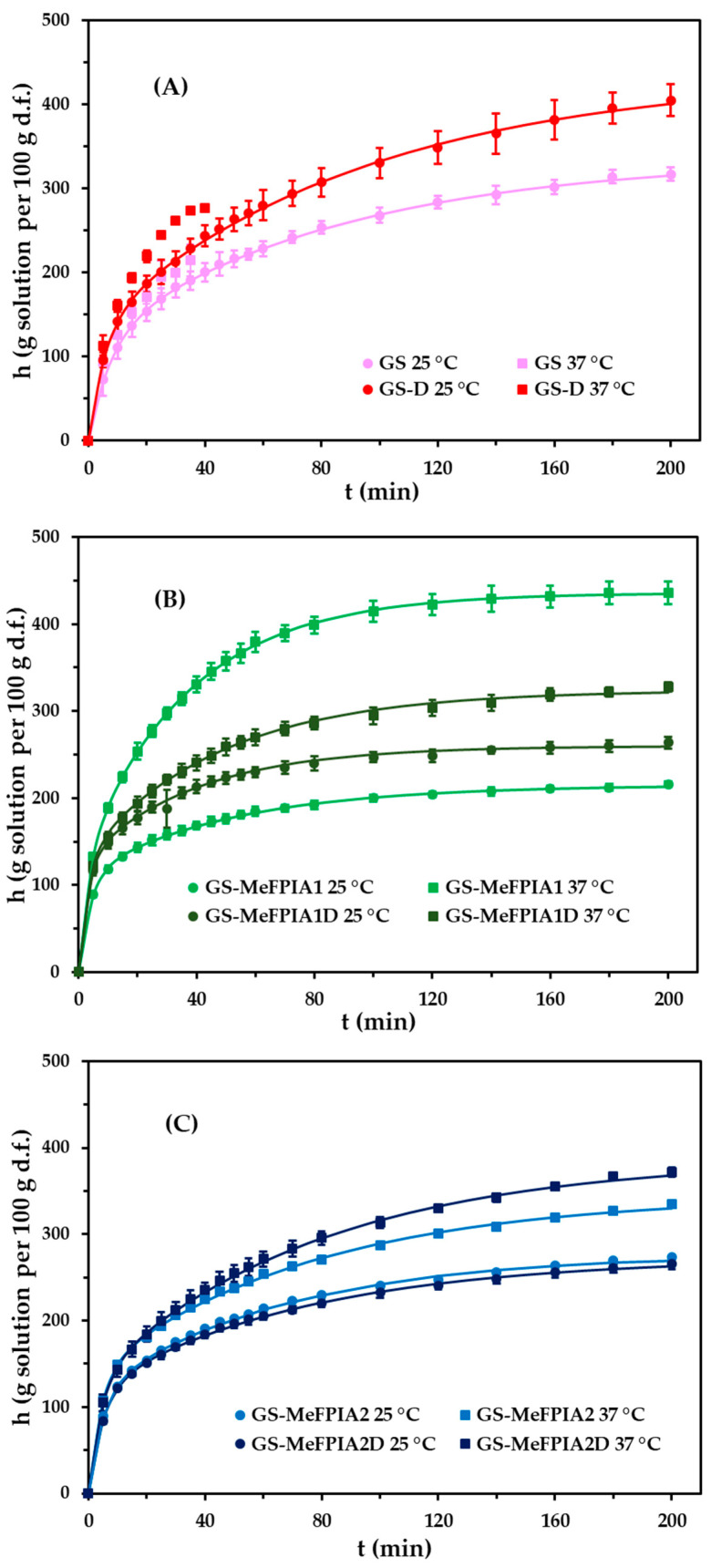
Swelling curves of the different films at 25 °C and 37 °C in phosphate buffer solution (PBS) buffer solutions: (**A**) GS and GS-D, (**B**) GS-MeFPIA1 and GS-MeFPIA1D, and (**C**) GS-MeFPIA2 and GS-MeFPIA2D.

The knowledge of the water or a solution transport towards the interior of some materials during swelling presents significant importance in different fields, such as medicine (drug delivery systems), environment (removal of contaminants), and agriculture (fertilizer delivery). Figure 4 displays the swelling behavior for the different films in PBS at 25 °C and 37 °C.

Control films showed the highest degree of swelling, as can be seen in Figure 4A, but they were the weakest matrices. Swelling of native gelatin-based films cannot be easily determined because of slow dissolution in water [62]. Over time, those films lost their ability to swell as well as their integrity, being dissolved in the media, as occurred at 37 °C, where the swelling process could not be concluded. Hence, cross-linking agents are necessary to introduce covalent bonds into the network, preventing dissolution of the gels [62]. Swelling capacity depends on many factors, such as the properties of the swelling liquid (pH, temperature, nature, and concentration of salts and properties, among others) and properties of the matrix (gelatin/starch ratio) itself (degree of degradation, presence of impurities, and conditions under which gelatin films were dried, among others) [63].

Films with MeFPIA1 and MeFPIA2 shown in Figure 4B,C, respectively, presented a lower degree of swelling compared with the control; in all the tested conditions, they were more stable, owing to the appearance of new interactions, mainly because of the presence of free carboxyl groups. It should be noted that, during the swelling test, the solubility of some of the components might have occurred, and this could be the case of dopamine. At the beginning, its release was not too fast, because the solution absorption rate was higher than the release of the additive; however, as time progressed, dopamine was slowly released to the media. It is possible that, as dopamine was released to the medium, free sites on the polymer chains appeared, able to interact with solution molecules, and retained it. In this study, samples containing dopamine better preserved their integrity during the test time. Although dopamine did not fully crosslink with polymer chains, as expected, because it was released into the swelling medium, it increased the number of bonds, especially with MeFPIAx, which contains carboxyl and ammonium groups to interact, providing greater stability [56].

The conversion temperature for gelatin is determined as melting point (gel to sol process), and the gel is converted into a solution as the temperature rises from 30 °C to 40 °C [32]. Among all the variables examined, the temperature increase from 25 °C to 37 °C produced an increase in the swelling process. This effect was caused by a rapid approach to the gel melting point as the temperature was raised. Thus, films swelled at 37 °C were softer and weaker than films swelled at 25 °C, indicating a lesser resistance to swelling [56].

Moreover, the results showed that the crosslinking was favored with the addition of modified MeFPIAx polymers and dopamine as the number of interactions increased. Then, it could be seen that the addition of MeFPIAx not only improved the mechanical properties of gelatin, but also decreased the absorption capacity of the solution molecules during the swelling process.

It is well-established that, when the behavior of water or solution absorption in polymers is considered a truly Fickian diffusion, the plot of (*W_t_* − *W*_0_)/*W*_0_ versus *t*^1/2^ should be linear up to almost a 60% increase in hydrogel mass, irrespective of any dependence of the diffusion coefficient on the moisture concentration [64]. In this way, and according to Fick’s second law and considering one-dimensional diffusion, the solution content as a function of time can be fitted with the power law equation when n is equal to 0.5, and the solution transport follows Fickian diffusion.

(8) $$\frac{\left(W_{t}-W_{0}\right)}{W_{0}\;}=kt^{n}$$

**Figure 5:**
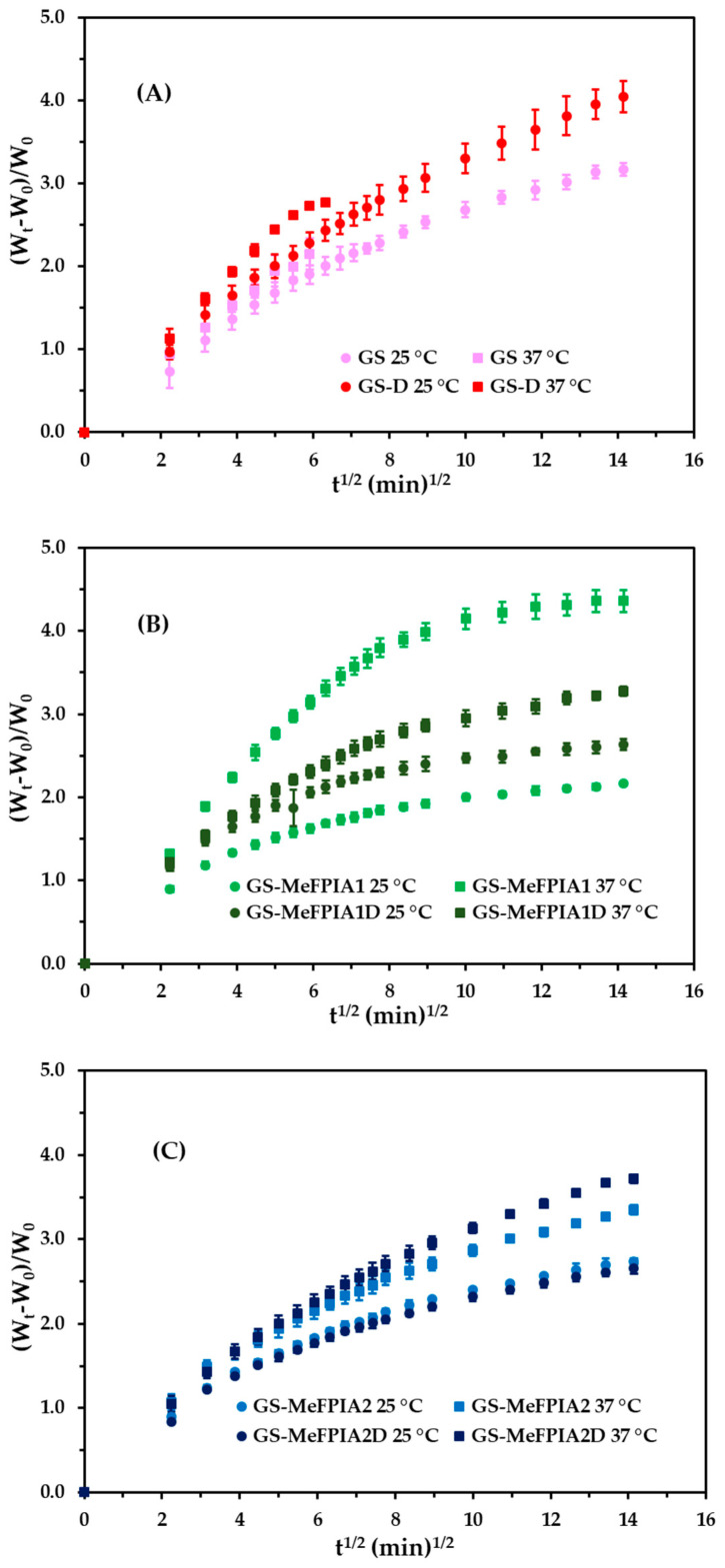
Representation of (*W_t_* − *W*_0_)/*W*_0_ versus *t*^1/2^ at 25 °C and 37 °C in PBS buffer solutions of (**A**) GS and GS-D, (**B**) GS-MeFPIA1 and GS-MeFPIA1D, and (**C**) GS-MeFPIA2 and GS-MeFPIA2D.

Consequently, and by plotting (*W_t_* − *W*_0_)/*W*_0_ versus *t*^1/2^ at initial stages of swelling, the Fickian behavior can be analyzed. The corresponding plots are displayed in Figure 5. As can be observed, the solution absorption curves of films for all the formulations were not linear up to a 60% increase in hydrogel mass. Therefore, as can be seen in the figure, most of the different formulations did not follow one-dimensional Fickian diffusion, so the values that n takes should be different to 0.5, and data should be adjusted by a non-Fickian or anomalous diffusion process.

It is described that dynamic factors, including rearrangements in the polymer structure, as a response to the sorption and diffusion process of solution, can be responsible for deviations from ideal Fick’s behavior. Indeed, these structural changes have their corresponding relaxation time constants. Consequently, the experimental data were fitted using a biexponential function in Equation (4). This equation expresses that the swelling process is produced through two different processes, one faster than the other. Data fitted quite well to the behavior of films (see lines of Figure 4).

The results of the different relaxation process that contributes to the solution uptake are collected in Table 5, where φ_1_ and φ_2_ are the fractions of solutions contributing to processes 1 and 2; this is Δh_1_/h_∞_ and Δh_2_/h_∞_ [65]. In all the curves, the square correlation was higher than 0.999, which indicates the adjustment accuracy. Table 5 shows that parameters for GS and GS-D at 37 °C were not determined (n.d.), because the films were disintegrated in the solution and could not be fitted.

The two processes, suggested by the biexponential model, can be attributed to a fast solution absorption mechanism more associated with the interaction of solution with the polymeric matrix, and a slow process related to the solution, less associated with the matrix, and that could be filling empty cavities and forming large multilayers of solution.

### 3.6. Antioxidant Activity

**Figure 6:**
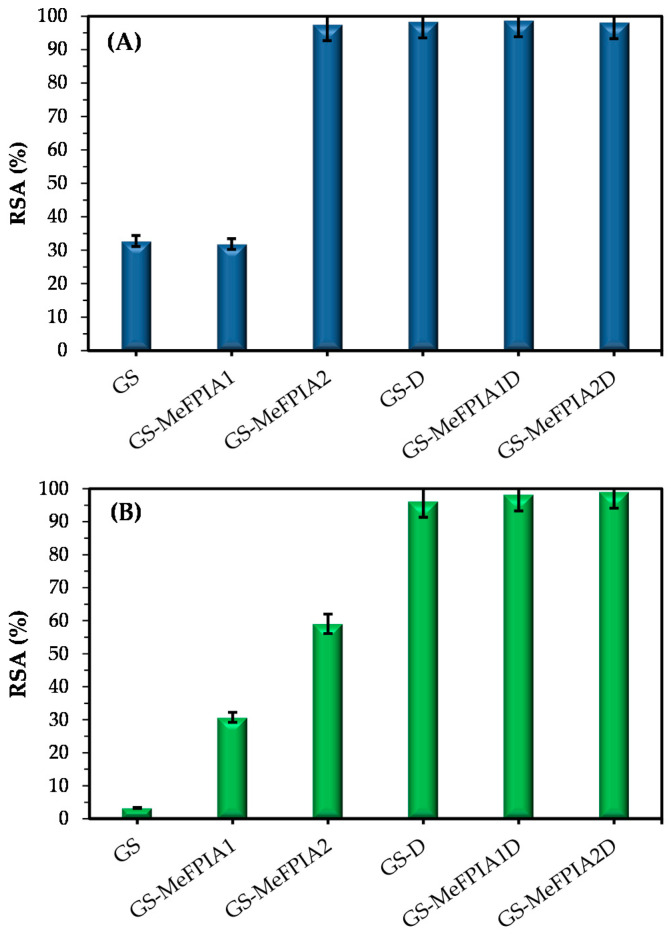

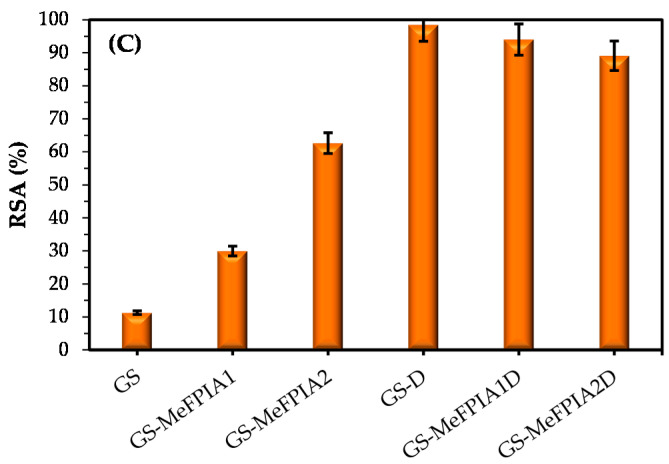
Antioxidant activity of gelatin-based films by the 2,2′-azino-bis(3-ethylbenzothiazoline-6-sulfonic acid) (ABTS) method after 30 min in (**A**) in water, (**B**) in ethanol/water 50/50, and (**C**) in ethanol/water 95/5. RSA, radical scavenging activity.

Figure 6 exhibits the results of antioxidant activity in different solutions. Based on the results, it could be seen that there was a decrease in the absorbance of the samples against control and, therefore, an increase in the RSA% value, caused by the antioxidant action of the developed films.

These values were even higher in films with dopamine, and an increase was seen with the increasing water content of solution B. It is well known that gelatin is soluble in water and in aqueous solutions of poly-hydric alcohols; however, it is practically insoluble in less polar organic solvents such as acetone, carbon tetrachloride, ethanol, ether, benzene, dimethylformamide, and most other nonpolar organic solvents [54]. Considering this, as the water content in solution B increased, so does the affinity with water, and samples swelled more rapidly, releasing dopamine more easily to the medium, increasing the antioxidant power of the dopamine. The increase in antioxidant activity in samples with dopamine added was because of the fact that many studies have indicated that dopamine was a strong free-radical inhibitor, which reacts with free radicals [42]. The dopamine molecule has a phenolic hydroxyl group in the structure (like most synthetic and natural antioxidants), and phenolic antioxidants have been recognized to function as hydrogen donors, acting like a free radical scavenger [66]. Surprisingly, when the assay was performed in solution B (water solution), the formulations with MeFPIA2 showed significant antioxidant activity even without the presence of dopamine. This fact could be because of a higher hydrogen donating potential of the network formed.

### 3.7. Biological Assays

#### 3.7.1. Antimicrobial Activity of MeFPIAx Quaternized Polymers

The MIC values are presented in Table 6 and they are the lowest concentrations (mg/mL) of the polymer that inhibit the growth of a microorganism after a 24 h incubation period at 37 °C [49]. MeFPIAx polymers were soluble in broth media and both presented antimicrobial activity. Previous studies have shown the key role of positive charge density of polycations and the amphiphilic balance in their interaction with the negatively charged surface of bacterial cell membranes, which, consequently, leads to cell death [24,67,68]. Moreover, the results of investigations supported the hypothesis that polymers bearing a cationic charge on the quaternary ammonium groups kill bacteria by damaging the cell membrane, causing cell lysis and exhibiting antimicrobial properties in solution and at the surfaces [69,70]. However, in this work, considering that a complete modification was not achieved because of the presence of the non-functionalized carboxyl groups, this situation influenced the decrease in the density of positive charges (as reflected in the ζ-potential), affecting the antimicrobial properties. MIC values for the antimicrobial polymers ranging from 0.5 to 8 mg/mL were obtained (see Table 6). Although there were no differences in MIC values against *Escherichia coli*, MeFPIA1 showed greater antimicrobial activity than MeFPIA2 against *Pseudomonas aeruginosa*. Many studies reported that molecular weight and spatial distribution of charge are other factors to consider on antimicrobial properties of charged polymers [68,71]. Hence, MeFPIA2 could adopt a conformation in the solution with the positive charge less accessible to the bacterial cell walls. Moreover, it is also described that anionic polymers and polyampholytes can present antimicrobial characteristics [22,72].

#### 3.7.2. Antimicrobial Activity of the Films

**Figure 7:**
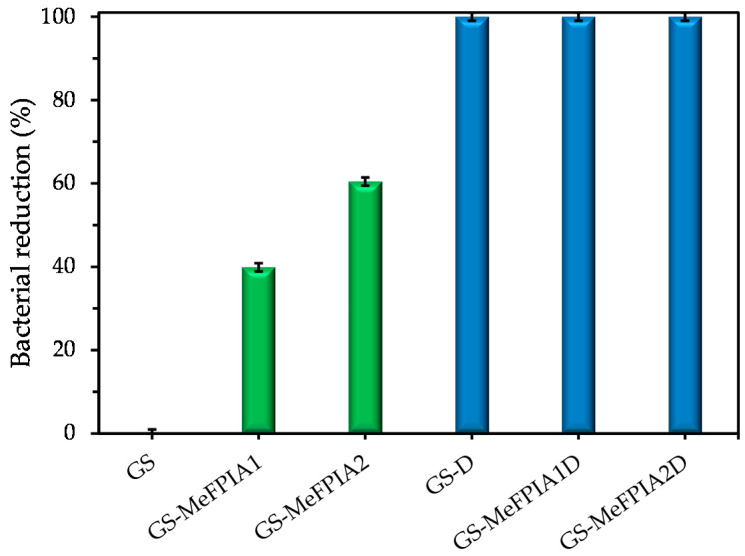
Bacterial reduction percentage against *S. aureus* bacteria of the different formulations.

The results presented in Figure 7 show the bacterial reductions (%) of the elaborated films after a 24 h incubation period at 37 °C with slight agitation in contact with *S. aureus* bacteria. Based on the reduction percentages obtained, it can be concluded that films with dopamine, GS-D, GS-MeFPIA1D, and GS-MeFPIA2D, showed complete inhibition due to the antimicrobial activity of the phenolic compound [55]. In addition, films containing MeFPIA1 and MeFPIA2 (without dopamine) also presented antimicrobial activity. This activity was even greater for those samples with MeFPIA2, as expected, because that polymer presented a higher degree of functionalization (quaternization), and more cationic groups are available at the film surface for contact killing behavior. The control samples, without MeFPIAx and without dopamine, did not show any inhibition. Koser et al. [73] studied the nutritional requirements of some bacteria, and indicated that gelatin was used by microorganisms as a growth-promoting source. Considering this and taking into account that the control films were the weakest and lost their integrity over time, as mentioned in Section 3.4, the microorganism surely used gelatin, as a nutrient for its growth, presenting a number of colony forming units greater than the initial one.

## 4. Conclusions

The preparation of active polymers was possible by conventional radical polymerization of IA, chemical modification of PIA incorporating thiazole groups at two different temperatures, and posterior quaternization reaction. The resulting modified polymers were monitored by ^1^H-NMR and ATR-FTIR, showing that the increase of temperature favors the incorporation of pendant heterocycles groups, and that the total quaternization of these groups was achieved in both cases. The ζ-potential also confirmed a greater degree of modification when the reaction temperature was increased. These polymers showed antimicrobial activity, exhibiting MIC values ranging from 0.5 to 8 mg/mL. The addition of MeFPIAx into gelatin-based films not only improved the mechanical properties of gelatin in dry state, but also decreased the absorption capacity of the water molecules during the swelling process. However, the increase in EB values due to the incorporation of polymer negatively affected the water vapor permeability, obtaining higher values compared with the control films. Additionally, those films reported antimicrobial and antioxidant activity compared with the control samples, and the addition of dopamine enhanced both properties. Furthermore, color changes were observed and were perceptible to the human eye; this change was more abrupt with the addition of dopamine.

In conclusion, the incorporation of MeFPIAx polymers and active agents such as dopamine into polymeric systems and, taking into account their properties, could be considered as good candidates to develop novel materials, expanding the functionality of food packaging.

## Figures and Tables

**Table 1:** Description of gelatin-based films.

Sample Names	Description
GS	Films based on gelatin, starch, and glycerol
GS-MeFPIA1	Films based on gelatin, starch, glycerol, and MeFPIA1
GS-MeFPIA2	Films based on gelatin, starch, glycerol, and MeFPIA2
GS-D	Films based on gelatin, starch, glycerol, and dopamine
GS-MeFPIA1D	Films based on gelatin, starch, glycerol, MeFPIA1, and dopamine
GS-MeFPIA2D	Films based on gelatin, starch, glycerol, MeFPIA2, and dopamine

**Table 2:** Mechanical parameters of gelatin-based films.

Formulations	Tensile Strength (MPa)	Young’s Modulus (Mpa)	Elongation at Break (%)
GS	2.86 ± 0.32 ^a^	15.7 ± 3.0 ^a^	140 ± 6 ^a^
GS-MeFPIA1	5.24 ± 0.26 ^c^	12.2 ± 2.4 ^abc^	199 ± 7 ^c^
GS-MeFPIA2	4.18 ± 0.51 ^b^	12.6 ± 4.8 ^abc^	170 ± 7 ^bd^
GS-D	3.07 ± 0.15 ^ad^	16.4 ± 2.0 ^a^	146 ± 10 ^a^
GS-MeFPIA1D	3.59 ± 0.29 ^d^	10.9 ± 4.0 ^bc^	183 ± 5 ^d^
GS-MeFPIA2D	3.32 ± 0.29 ^ad^	9.9 ± 1.9 ^bc^	166 ± 12 ^b^

The same letters in the data reported in a column mean non-significant differences (*p* ≥ 0.05).

**Table 3:** Color properties of gelatin-based films.

Formulations	L*	a*	b*	ΔL*	Δa*	Δb*	ΔE*
GS	89 ± 1 ^a^	1 ± 0.02 ^a^	−1 ± 0.1 ^a^				
GS-MeFPIA1	87 ± 1 ^a^	−3 ± 0.2 ^c^	18 ± 1 ^c^	−2	−4	18	19
GS-MeFPIA2	83 ± 1 ^c^	−3 ± 0.2 ^c^	34 ± 4 ^b^	−6	−4	35	36
GS-D	56 ± 3 ^b^	9 ± 1 ^b^	34 ± 1 ^b^	−33	7	35	49
GS-MeFPIA1D	62 ± 2 ^e^	8 ± 1 ^b^	37 ± 1 ^b^	−27	6	38	47
GS-MeFPIA2D	47 ± 1 ^d^	16 ± 0.4 ^d^	36 ± 2 ^b^	−42	14	37	58

The same letters in the data reported in a column mean non-significant differences (*p* ≥ 0.05).

**Table 4:** Experimental water vapor permeability values of gelatin-based films.

Formulations	*P_w_^exp^* (g·s^−1^·m^−1^·Pa^−1^)
GS	8.7 ± 0.1 ^b^
GS-MeFPIA1	11.1 ± 0.1 ^e^
GS-MeFPIA2	9.3 ± 0.1 ^c^
GS-D	8.4 ± 0.1 ^a^
GS-MeFPIA1D	9.9 ± 0.1 ^d^
GS-MeFPIA2D	8.5 ± 0.1 ^a^

The same letters in the data reported in a column mean non-significant differences (*p* ≥ 0.05).

**Table 5:** Kinetic parameters of swelling for the different gelatin-based films.

Formulations	Temp. (°C)	h_∞_ (g/g)	Δh_1_ (g/g)	τ_1_ (min)	φ_1_	Δh_2_ (g/g)	τ_2_ (min)	φ_2_
GS	25	336 ± 7	118 ± 4	7.6 ± 0.4	0.35	218 ± 3	85 ± 4	0.65
GS-MeFPIA1		215 ± 5	109 ± 3	4.0 ± 0.2	0.51	106 ± 2	50 ± 2	0.49
GS-MeFPIA2		277 ± 7	114 ± 4	4.6 ± 0.3	0.41	163 ± 3	65 ± 3	0.59
GS-D		436 ±10	132 ± 6	5.7 ± 0.4	0.30	304 ± 4	93 ± 5	0.70
GS-MeFPIA1D		260 ± 11	123 ± 6	3.0 ± 0.4	0.47	137 ± 5	38 ± 3	0.53
GS-MeFPIA2D		273 ± 5	117 ± 3	5.1 ± 0.3	0.43	156 ± 2	73 ± 3	0.57
GS	37	n.d.	n.d.	n.d.	n.d.	n.d.	n.d.	n.d.
GS-MeFPIA1		436 ± 7	118 ± 4	3.4 ± 0.2	0.27	318 ± 3	36 ± 1	0.73
GS-MeFPIA2		345 ± 7	137 ± 4	4.5 ± 0.3	0.40	208 ± 3	76 ± 4	0.60
GS-D		n.d.	n.d.	n.d.	n.d.	n.d.	n.d.	n.d.
GS-MeFPIA1D		324 ± 10	125 ± 6	3.1 ± 0.4	0.39	199 ± 4	46 ± 2	0.61
GS-MeFPIA2D		388 ± 8	129 ± 5	4.6 ± 0.4	0.33	259 ± 3	78 ± 4	0.67

**Table 6:** Minimal inhibition concentration (MIC) values (mg/mL) of the quaternized polymers MeFPIA1 and MeFPIA 2.

Polymers	MIC (mg/mL)		
	*E. coli*	*P. aeruginosa*	*S. aureus*
MeFPIA1	8	2	0.5
MeFPIA2	8	4	0.5

## Data Availability

Data sharing not applicable.
